# Contemporary assessment of diagnostic performance and histologic concordance of renal mass biopsy with surgical pathology

**DOI:** 10.1002/bco2.70104

**Published:** 2025-11-06

**Authors:** Mitchell M. Huang, Cristina B. Arruza‐Frau, Austin P. Drysch, Nicole Handa, Ridwan Alam, Behtash G. Nezami, Ashley E. Ross, Niraj K. Shenoy, Kent T. Perry, Hiten D. Patel

**Affiliations:** ^1^ Department of Urology, Feinberg School of Medicine Northwestern University Chicago IL USA; ^2^ Robert H. Lurie Comprehensive Cancer Center Feinberg School of Medicine Chicago IL USA; ^3^ Department of Pathology, Feinberg School of Medicine Northwestern University Chicago IL USA; ^4^ Surgery Service Jesse Brown VA Medical Center Chicago IL USA

**Keywords:** biopsy, histology, nephrectomy, renal mass

## Abstract

**Background:**

Improvements in imaging and technique have led to a greater role for renal mass biopsy (RMB) to risk‐stratify localized renal masses. We sought to assess the contemporary diagnostic accuracy of RMB for identifying renal cell carcinoma (RCC) based on concordance with final surgical pathology.

**Materials and Methods:**

Consecutive patients undergoing RMB between 2013 and 2023 across an 11‐hospital health system were identified. Concordance between RMB and final pathology for patients receiving partial or radical nephrectomy was compared. We calculated sensitivity, specificity, positive predictive value (PPV) and negative predictive value (NPV) of RMB for identifying RCC among patients who underwent surgery.

**Results:**

A total of 733 patients underwent RMB with an overall non‐diagnostic rate of 11% and most biopsies (67%) identified RCC. A total of 243 patients with surgical pathology were analysed, and 229 (94%) had RCC on surgical pathology. Excluding non‐diagnostic cases, RMB had a 98% concordance with surgical pathology for RCC; RMB had 94% sensitivity, 71% specificity, 98% PPV and 42% NPV for identifying RCC with non‐diagnostic cases considered as benign biopsies. For the nine RMB oncocytic cases receiving surgery, six (66%) were RCC and only two (22%) were confirmed oncocytomas. For patients with biopsy grade reported, 100% of high‐grade RCC were confirmed while 26% of low‐grade RCC were upgraded at nephrectomy (overall 80% concordance).

**Conclusions:**

In a large, contemporary cohort of patients, RMB had high PPV and sensitivity for identifying RCC and high concordance for specific histology at nephrectomy. However, upgrading was common, and oncocytic lesions selected for surgery due to clinical suspicion often harboured RCC.

## 1 INTRODUCTION

Kidney cancer is diagnosed in over 80 000 people and causes more than 14 000 deaths annually in the United States.^1^ The incidence of renal cancer is rising due to widespread use of cross‐sectional imaging.^2^ Most new diagnoses are small, localized renal masses with a high likelihood of being indolent.^3^, ^4^, ^5^ Upwards of 6000 renal masses removed each year are benign on final pathology.^6^ Renal mass biopsy (RMB) can help avoid unnecessary surgery by identifying benign masses, while also aiding potential risk stratification and management decisions for malignant tumours.^7^, ^8^, ^9^

The American Urological Association (AUA) guidelines recommend RMB when the outcome may influence management, with exceptions for younger patients who are not comfortable with the uncertainties of RMB and older patients who will be managed conservatively regardless of the result.^10^ However, there remains provider‐to‐provider variation in the utilization of RMB, some of which may be driven by concerns about the diagnostic accuracy of RMB.^11^, ^12^, ^13^ Recent estimations of RMB utilization have reported wide practice‐based variation, and despite increased rates of biopsy, currently fewer than 20% of patients with small renal masses (SRMs) undergo RMB. Despite concerns about the safety and diagnostic yield of RMB, retrospective data suggest RMB is a safe procedure with reasonable sensitivity and specificity for identifying malignancy.^12^, ^14^, ^15^ Biopsy has also been shown to affect downstream treatment decisions, with biopsied patients less likely to undergo surgery in favour of active surveillance.^8^, ^16^

In this study, we assessed the diagnostic performance of RMB using contemporary data from a large, multi‐hospital health system. The primary aim was to evaluate histologic concordance between RMB and nephrectomy focusing on outcomes of identifying (1) any renal cell carcinoma (RCC) and (2) specific histologic subtypes. Secondary aims included identifying the overall non‐diagnostic rate for RMB, evaluating the subgroup of oncocytic tumours receiving surgery due to clinical suspicion, and assessing the identification of RCC grade on biopsy.

## 2 MATERIALS AND METHODS

### 2.1 Biopsy cohort

We identified all patients who underwent RMB for a renal mass between the years of 2013 and 2023 across an 11‐hospital health care system (Northwestern Medicine, Chicago, IL) using the Northwestern Medicine Enterprise Data Warehouse (EDW). We obtained institutional review board approval to review de‐identified clinical information for these patients. Baseline demographic and clinical characteristics, including age, sex, race, BMI and relevant medical comorbidities were included in our analysis. We excluded patients who underwent biopsy for medical renal disease. For the overall cohort of biopsied patients, we tabulated baseline clinical characteristics and calculated a non‐diagnostic rate. For the subgroup with oncocytic lesions noted on biopsy (defined as oncocytoma, oncocytic neoplasm or chromophobe RCC), we identified the frequency of immunohistochemical staining for CK7 and CD117, and the rate of subsequent surgical management.

### 2.2 Biopsy to surgical pathology concordance

For biopsy to surgical pathology concordance, we identified the subset of patients subsequently receiving radical or partial nephrectomy (including open, hand‐assist, laparoscopic and robotic‐assisted approaches). Our primary aim was to identify overall histologic concordance between biopsy and final surgical pathology for (1) any RCC and (2) specific histologic subtype. Sensitivity, specificity, positive predictive value (PPV) and negative predictive value (NPV) were calculated for RMB to predict cancer of any type (RCC or otherwise) as well as RCC (irrespective of subtype) on nephrectomy. Of note, we included clear cell papillary renal cell tumours as a benign entity, separate from RCC.^17^ We analysed final histologic outcomes for patients with oncocytic lesions on biopsy receiving surgery due to clinical suspicion. Given concerns about the diagnostic yield of RMB for small renal masses (cT1a, ≤4 cm in size), we stratified our analysis to the ≤4 cm subgroup based on pathologic lesion size. Lastly, we compared biopsy grade to final nephrectomy grade; grade was grouped into low (grade 1 or 2) and high (grade 3 or 4) to determine sensitivity, specificity, PPV and NPV of biopsy grade.

### 2.3 Statistical analysis

All statistical analysis was performed using R version 4.4.2 (R Project for Statistical Computing) and the “dplyr” package to code and process the data. Tables and figures were generated using Microsoft Excel (Microsoft Corporation, Redmond, WA). Bivariate statistical comparisons were made using the Wilcox rank‐sum and Fisher's exact test as appropriate using a cutoff of *P* < 0.05 for statistical significance. Diagnostic performance was measured by calculating sensitivity, specificity, PPV and NPV.

## 3 RESULTS

### 3.1 Biopsy cohort

**FIGURE 1:**
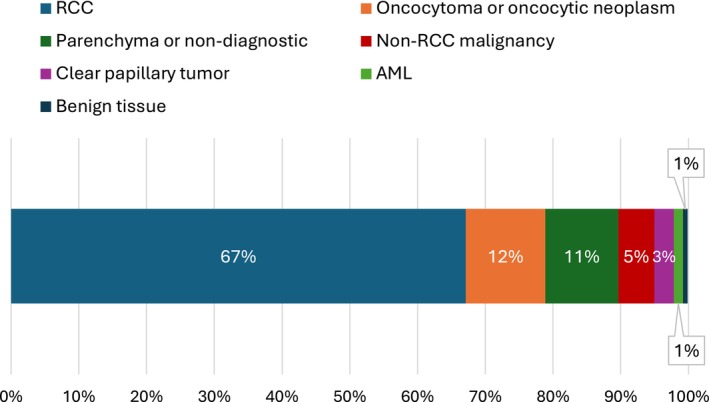
Breakdown of histologic findings for the overall biopsy cohort (n = 733). **Abbreviations**: RCC – renal cell carcinoma, AML – angiomyolipoma. “RCC” group includes all subtypes of RCC, “oncocytoma or oncocytic neoplasm” group does not include chromophobe RCC. “Benign tissue” group defined as either haemangioma, confirmed cyst or inflammatory tissue.

We identified a total of 733 patients who underwent RMB for renal masses suspicious for RCC. Patient characteristics are summarized in Table 1. The cohort had a median age of 72 years (IQR 64–80) and consisted of 266 (36%) female patients and 215 (29%) non‐white patients. The median BMI was 29.5 (IQR 25.6–34.1), and the majority of biopsies were core biopsies (657, 90% core vs. 76, 10% fine needle aspiration). For the overall cohort of biopsied patients, the non‐diagnostic rate was 11% (79/733). Of the biopsied patients, 492 (67%) had RCC including 310 (42%) clear cell, 86 (12%) oncocytoma or oncocytic neoplasms, 39 (5%) non‐RCC malignancies and 10 (1%) angiomyolipoma (AML) (Table 2, Figure 1). Of the 492 patients with RCC on biopsy, 227 (46%) underwent surgery, 114 (23%) ablative treatment, 21 (4%) treatment for metastatic disease and 130 (26%) underwent surveillance or no treatment. Of the 86 patients with oncocytoma or oncocytic masses (excluding definitive chromophobe RCC) on biopsy, 13 (15%) underwent focal ablation, 9 (10%) surgery, 3 (3%) treatment for other non‐renal metastatic disease and the remaining 61 (71%) underwent surveillance or no treatment. Of the 21 clear cell papillary renal cell tumours on biopsy, 8 (38%) underwent surgery, 5 (24%) focal treatment, 1 (5%) treatment for non‐renal metastatic disease and 7 (33%) underwent surveillance or no treatment.

**TABLE 1:** Baseline characteristics of overall biopsy cohort and the subset who underwent nephrectomy.

	Biopsy only (n = 490)	Nephrectomy (n = 243)	Overall (n = 733)	*P*‐value
**Age, median (IQR)**	73 (66–81)	69 (62–76)	72 (64–80)	<0.001
**Sex, n (%)**				0.4
Male	307 (63)	160 (66)	467 (64)	
Female	183 (37)	83 (34)	266 (36)	
**Race, n (%)**				0.5
Asian	8 (2)	8 (3)	16 (2)	
Black	75 (15)	41 (17)	116 (16)	
Latino/Latina	22 (4)	6 (2)	28 (4)	
Other	30 (6)	25 (10)	55 (7)	
White	355 (72)	163 (67)	518 (71)	
**BMI, median (IQR)**	30.1 (25.7–34.4)	28.8 (25.2–33.5)	29.5 (25.6–34.1)	0.05
**Mass size on imaging, median (IQR)**	2.9 (2.0–3.9)	3.8 (2.4–6.8)	3.4 (2.3–5.6)	<0.001

**Abbreviations**: BMI = body mass index.

**TABLE 2:** Breakdown of biopsy histology for complete cohort (n = 733).

Histology	n	(%)
**RCC**	492	67%
Clear cell	310	42%
Papillary	112	15%
Chromophobe	33	5%
Sarcomatoid/rhabdoid	4	1%
Unspecified	33	5%
**Oncocytoma/Oncocytic**	86	12%
**Clear papillary tumour**	21	3%
**AML**	10	1%
**Parenchyma/Non‐diagnostic**	79	11%
**Benign** *	5	1%
**XGP**	1	0%
**Other cancer**	39	5%
Lymphoma	17	2%
Necrotic or poorly differentiated	10	1%
Urothelial	5	1%
Plasma cell	2	0%
Squamous cell carcinoma	1	0%
Collecting duct carcinoma	1	0%
Lung adenocarcinoma	1	0%
Spindle cell mass	1	0%
Metanephric malignancy	1	0%

* Benign cyst, haemangioma or inflammatory tissue.

**Abbreviations**: RCC ‐ renal cell carcinoma; AML ‐ angiomyolipoma; XGP – xanthogranulomatous pyelonephritis.

### 3.2 Biopsy to surgical pathology concordance

Overall, 243 patients from the initial RMB sample underwent surgery in our system of which 158 (65%) were partial nephrectomies and 85 (35%) were radical nephrectomies. Compared to the rest of the biopsy cohort, the surgical group was younger (median 69, IQR 62–76 vs 73, IQR 66–81*, P* < 0.001) and trended towards lower BMI (28.8, IQR 25.2–33.5 vs. 30.1, IQR 25.7–34.4, *P* = 0.05). The median tumour size on imaging was 3.8 cm (2.4–6.8) and 4.1 cm on surgical specimens (IQR: 2.8–6.4 cm). Of note, oncocytoma and oncocytic masses selected for surgery due to clinical suspicion were smaller than those not selected for surgery (median 1.5 cm, IQR 1.3–1.6 vs. 2.5 cm, IQR 2.0–3.9 for oncocytoma and oncocytic masses that did not go to surgery, *P* < 0.001); this suggested that larger size was not the primary indication for surgery for these masses. Reasons for pursuing surgery for oncocytoma/oncocytic neoplasm on biopsy included CK7 positivity (three cases), interval growth of the tumour or concerning growth kinetics (3), and concerns about under‐sampling or uncertainty in pathologic diagnosis (3).

In the surgical sample, RMB had a non‐diagnostic rate of 5% based on the presence of benign parenchyma. Treating 11 non‐diagnostic biopsies as true benign results, the concordance of RMB and nephrectomy for RCC (considering clear cell papillary renal cell tumour as non‐RCC) and exact histology was 98% (215/219) and 88% (214/243), respectively. RMB had a sensitivity of 94% (215/229), specificity of 71% (10/14), PPV of 98% (215/219) and NPV of 42% (10/24) for identifying RCC (ignoring the specific subtype, considering “oncocytic neoplasm” on biopsy as RCC and considering clear papillary tumour as non‐RCC) (Supplemental Table 1).

Excluding the 11 non‐diagnostic cases from performance measures, RMB had the same concordance of 98% (215/219) for identifying RCC, but a higher concordance for exact histology of 92% (214/232). When excluding the non‐diagnostic biopsy cases, RMB had a sensitivity of 98% (215/219), specificity of 69% (9/13), PPV of 98% (213/217) and NPV of 69% (9/13) for identifying RCC, ignoring the specific subtype (Supplemental Table 2).

### 3.3 Individual histology

**FIGURE 2:**
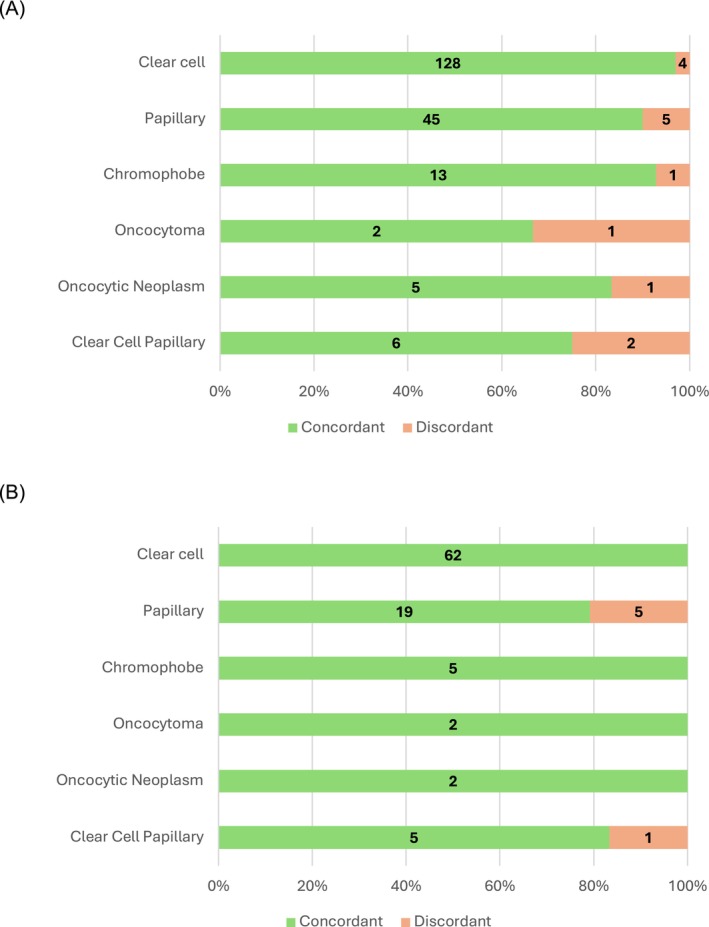
Concordance between surgical and biopsy histology for overall biopsy cohort (a.) and for subset with masses <4 cm (b.). listed histology subtype is based on biopsy, with colouring indicating the proportion with concordance/discordance on surgical pathology.

**FIGURE 3:**
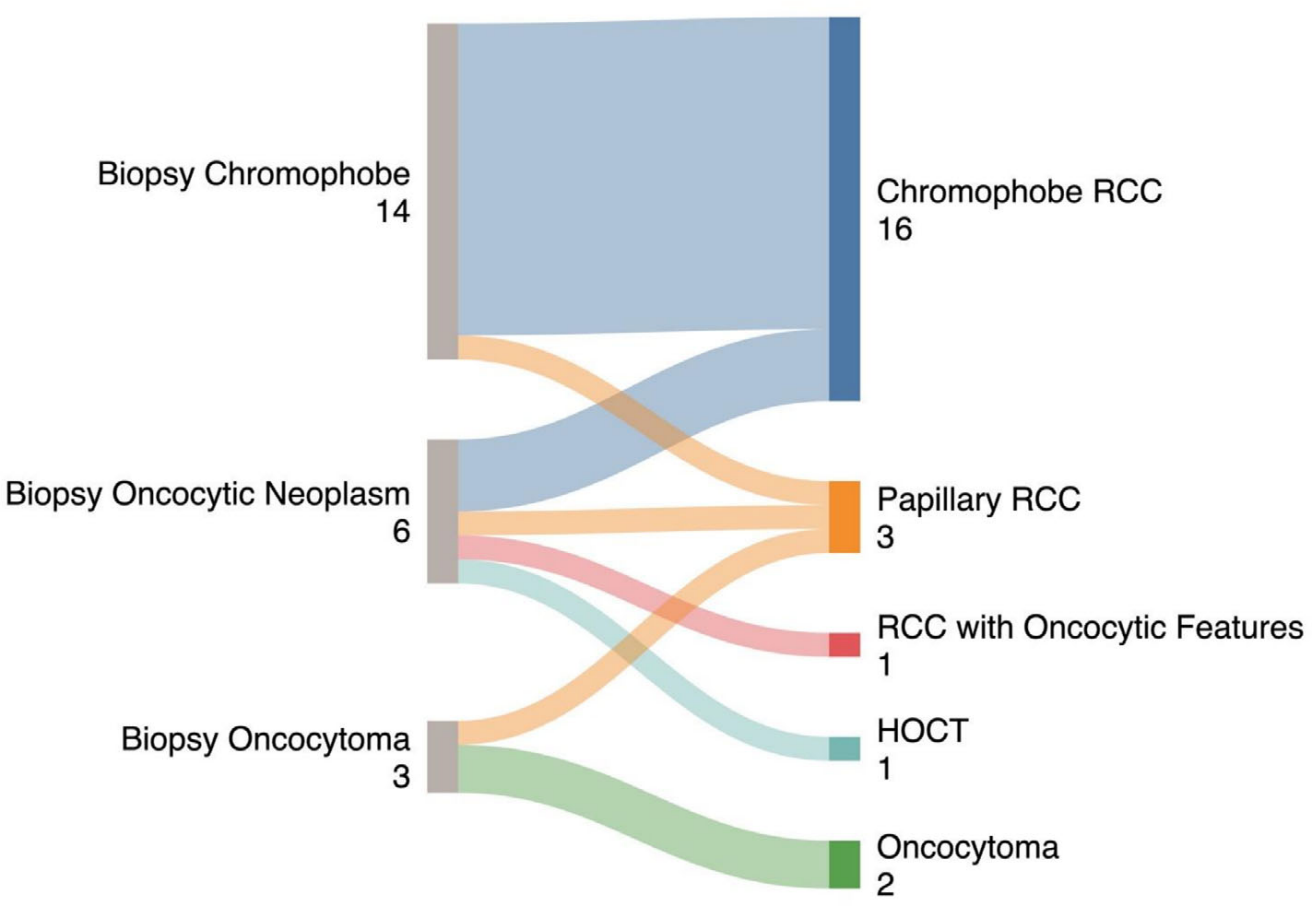
Breakdown of final subtype on surgical subtype for the 23 patients with oncocytic neoplasm, oncocytoma or chromophobe RCC on biopsy. **Abbreviations:** RCC – renal cell carcinoma, HOCT – hybrid oncocytic/chromophobe tumour.

The most common histology on both biopsy and nephrectomy was clear cell RCC (54% biopsy, 59% nephrectomy) followed by papillary RCC (21% biopsy, 24% nephrectomy) and chromophobe RCC (6% biopsy, 7% nephrectomy) (Table 3). These distributions were similar for the small renal mass cohort (*P* = 0.7). The concordance rates by individual subtype were high, with 97% (128/132) of clear cell RCC on biopsy confirmed on surgery (Figure 2a). 90% (45/50) of papillary RCC and 93% (13/14) of chromophobe RCC on biopsy were confirmed on nephrectomy. These concordance rates remained high when examining the small mass cohort (Figure 2b). Of the 118 patients in the overall biopsy cohort who had oncocytic lesions (chromophobe RCC, oncocytoma or oncocytic masses), 67 (57%) had CK7 staining and 57 (48%) had CD117 staining reported on their pathology reports. Of these 118 patients, 23 (19%) underwent surgery and the majority (19, 83%) were RCC on nephrectomy (Figure 3), including 16 (70%) chromophobe RCC. Excluding the chromophobe RCC cases on biopsy, 9 oncocytomas or oncocytic neoplasms on biopsy underwent surgery with only 2 (22%) confirmed oncocytoma on final pathology with others showing chromophobe (3, 33%), hybrid oncocytic tumour (1, 11%), papillary RCC (2, 22%) and RCC with oncocytic features (1, 11%).

**TABLE 3:** Comparison of histology distribution between biopsy and nephrectomy for the surgery cohort.

	Biopsy (n = 243)	Nephrectomy (n = 243)	Biopsy small mass (≤4 cm) (n = 111)	Nephrectomy small mass (≤4 cm) (n = 111)
**Renal cell carcinoma**	213 (88%)	229 (94%)		
Clear cell	132 (54%)	142 (58%)	62 (56%)	69 (62%)
Papillary	50 (21%)	59 (24%)	24 (22%)	23 (20%)
Chromophobe	14 (6%)	17 (7%)	5 (5%)	6 (5%)
Sarcomatoid	2 (1%)	0 (0%)	‐	‐
Unspecified	15 (6%)	4 (2%)	3 (3%)	1 (1%)
Other RCC*	‐	7 (3%)		4 (4%)
**Oncocytic neoplasm** **	6 (2%)	1 (<1%)	2 (2%)	0 (0%)
**Clear cell papillary**	8 (3%)	8 (3%)	6 (5%)	5 (5%)
**Oncocytoma** **	3 (1%)	2 (1%)	2 (2%)	2 (2%)
**Parenchyma**	11 (5%)	1 (<1%)	7 (6%)	0 (0%)
**Other**	2 (1%)+	2 (1%)^§^	0 (0%)	1 (1%)

* includes TFE‐rearrangement, fumarate‐hydratase deficient, oncocytic features.

** of the nine biopsy oncocytoma or oncocytic neoplasm cases on biopsy, three (33%) were confirmed to be oncocytoma on surgery.**

+includes angiomyolipoma and xanthogranulomatous pyelonephritis.

^§^ includes cavernosal haemangioma and lymphoproliferative disorder.

**Abbreviations:** RCC – renal cell carcinoma.

### 3.4 Grade concordance

Grade was reported on 108 of the 219 RCC biopsy cases (49%). Of the 109 surgical patients overall who had biopsy grade reported (the additional 1 case with biopsy grade reported was a clear cell papillary tumour), 87 (80%) had concordant grade on nephrectomy. Overall, 22/84 (26%) of patients upgraded from low‐ to high‐grade on nephrectomy while all high‐grade biopsies were confirmed (25/25). The overall sensitivity, specificity, PPV and NPV of biopsy grade for predicting final grade on nephrectomy were 53%, 100%, 100% and 74% (Table 4). For the small mass cohort, the overall rate of grade concordance was similar (84%, 46/55, *P* = 0.7); sensitivity, specificity, PPV and NPV of biopsy grade were 40%, 100%, 100% and 82% (*P* = 0.6, 1.0, 1.0 and 0.4, respectively, compared to the overall cohort).

**TABLE 4:** Comparison of biopsy grade (low vs. high) by biopsy and nephrectomy pathology.

		Nephrectomy grade, n (%)	
		Low (1–2)	High (3–4)
**Biopsy Grade, n (%)**	Low (1–2)	62 (57)	22 (20)
	High (3–4)	0 (0)	25 (23)

## 4 DISCUSSION

Although many renal tumours are indolent and do not need immediate treatment, RMB could help identify more aggressive tumours and guide downstream management decisions. While RMB utilization has increased in recent years, estimates suggest that only 15–16% of patients with SRMs undergo biopsy and that there is significant provider‐level variation in this figure.^11^, ^12^, ^15^ Some hesitation may be due to concerns about the diagnostic accuracy of RMB. In this study, we provided an updated and detailed assessment of RMB's predictive performance in a contemporary cohort. Overall, we report a moderate non‐diagnostic rate of 11%, but an overall concordance rate of 88% for exact histology based on patients who underwent nephrectomy. We found that although the sensitivity (94%) and PPV (98%) of RMB for identifying RCC were high, the specificity (71%) and the NPV (42%) were poor reflecting the accurate selection of benign biopsy for intervention based on clinical suspicion. Furthermore, we found that grade was reported in half of RCC cases. The overall concordance of biopsy grade and nephrectomy grade was 80%, with good specificity and PPV (both 100%) for identifying high‐grade cancer but poor sensitivity (53%) and NPV (74%).

These findings demonstrate the contemporary performance of RMB with detailed consideration of specific histologic subtypes and advances in knowledge of indolent tumour subtypes including clear cell papillary tumours. A large systematic review of 20 studies reviewed 3113 biopsies and reported an overall non‐diagnostic rate of 14% and a false‐positive rate of 4%. They noted that biopsy had a high concordance of histologic subtype when compared to nephrectomy but reported substantial rates of upgrading (16%).^14^ These figures are comparable to the rates identified in our study, with the exception of NPV, which was notably lower in our study (42%) than previously reported rates. The largest pooled estimate of RMB's NPV was 68.5%^14^ to identify malignancy and the previously lowest reported NPV was 50.5% in a cohort of 42 patients undergoing RMB.^18^ The lower NPV observed in our study is likely due to how we chose to classify “negative” biopsies in our primary analysis. Notably, our NPV was 69% when the 11 non‐diagnostic cases were excluded from analysis, which is consistent with the largest pooled estimate of NPV to date. Although previous studies have typically excluded non‐diagnostic cases from analysis, we treated them as “negative” biopsies in our study as we believe this to be a more clinically informed classification system. We believe that there is value in this observation of lower NPV when including “negative” biopsies—those who undergo nephrectomy despite a benign or indolent RMB result are more likely to have persistently suspicious tumours. This finding also suggests that providers at our institution have improved their selection of candidates for extirpative surgery over time through better interpretation of imaging and tumour characteristics. In addition, concordance between grade on biopsy and nephrectomy was high, but 26% of cases upgraded from low‐grade on biopsy to high‐grade on nephrectomy. Several studies have suggested that intratumoural heterogeneity may account for the difficulties in accurately predicting tumour grade on biopsy.^19^, ^20^

Accurately distinguishing oncocytoma from chromophobe RCC is an ongoing challenge in the management of renal masses. One review found that the PPV for oncocytoma on RMB was 67% based on the 22.4% of patients with oncocytic neoplasms on biopsy that eventually underwent surgery.^21^ These figures differ from our study where biopsy had a 22% PPV for oncocytoma based on 19% of patients with oncocytic neoplasms (including chromophobe RCC) receiving surgery. Of note, the majority of patients in our surgery cohort who had an oncocytic mass on biopsy were found to have RCC (usually chromophobe subtype). In other words, lesions suspicious for chromophobe RCC on biopsy were most often confirmed on surgery and the small subset of indeterminate oncocytic lesions selected for surgery were typically found to be RCC. This finding should be interpreted with caution, as only 10% of all biopsied non‐chromophobe oncocytic neoplasms were ultimately selected for surgery. However, our findings do suggest that when clinical suspicion is sufficiently high—such as when CK7 staining is positive, when there are concerns about under‐sampling, or when there is interval growth of a tumour—certain oncocytic masses may be appropriate for surgery. Although the recent advent of assays such as chromosomal analysis and next‐generation sequencing has demonstrated the ability to confirm chromophobe RCC,^22^, ^23^ traditional immunohistochemistry panels remain an important diagnostic tool, with CK7, CD117 and Claudin‐7 staining showing upwards of 94% specificity for chromophobe RCC in previous studies, although Claudin‐7 is typically not used in clinical practice anymore.^24^, ^25^ These tools can be further combined with radiomics or nuclear medicine imaging to improve selection for surgery.^26^, ^27^ Our study found that only half of biopsied patients had CK7 and CD117 staining, demonstrating that there is pathologist‐level variation in diagnostic confidence and changes in practice patterns surrounding the utilization of immunohistochemistry over time.

While our study included a large and recent cohort of biopsied patients across multiple hospitals, there are several limitations to note. First, it is a retrospective study and includes only patients within a single hospital system which may limit the generalizability to other institutions. Our cohort is predominantly white and several racial groups (in particular, Asian and Latino/Latina patients) were underrepresented when compared to their proportion in the general United States population. Furthermore, as with all studies evaluating concordance between biopsy‐surgical histology, we used surgical pathology as the gold standard for our analysis. As such, our estimations of biopsy accuracy are impacted by patients with benign pathology on biopsy and low suspicion for harbouring higher‐risk disease not undergoing nephrectomy. Overall, our findings demonstrate that RMB continues to perform well with a high positive predictive value for identifying RCC and high concordance with surgical pathology despite limitations due to oncocytic lesions.

## 5 CONCLUSIONS

In a cohort of patients who underwent RMB in the past decade, we found RMB had a moderate non‐diagnostic rate of 11% but a high positive predictive value (97%) for identifying RCC and high concordance for specific histology (88%) at nephrectomy. Although concordance for grade was reasonably high, the rate of low to high upgrading on nephrectomy was 26% with poor sensitivity and NPV of biopsy for identifying high‐grade disease. Only one in ten patients with oncocytic neoplasms on biopsy were clinically selected for surgery, but the majority of these had RCC on nephrectomy. Overall, these findings suggest that RMB continues to have high diagnostic accuracy in subtyping and identifying cancer and should continue to be an important tool in the diagnosis and management of renal masses when it may impact management decisions.

## AUTHOR CONTRIBUTIONS

**Mitchell M. Huang:** Conceptualization; data curation; formal analysis; methodology; validation; visualization; writing—original draft preparation; writing—review and editing. **Cristina B. Arruza‐Frau:** Formal analysis; visualization; writing—original draft preparation; writing—review and editing. **Austin P. Drysch:** Formal analysis; validation; writing—review and editing. **Nicole Handa:** Supervision; investigation; writing—review and editing. **Ridwan Alam:** Supervision; validation; writing—original draft preparation; writing—review and editing. **Behtash G. Nezami:** Supervision; validation; writing—review and editing. **Ashley E. Ross:** Resources; project administration; writing—review and editing. **Niraj K. Shenoy:** Supervision; validation; writing—review and editing. **Kent T. Perry:** Supervision; methodology; validation; writing—review and editing. **Hiten D. Patel:** Conceptualization; data curation; formal analysis; validation; supervision; funding acquisition; writing—original draft preparation; writing—review and editing.

## CONFLICT OF INTEREST STATEMENT

None.

## Supporting information


**Supplemental Table 1.** Sensitivity, specificity, PPV, NPV for identifying renal cell carcinoma (includes 11 non‐diagnostic/parenchyma biopsy).
**Supplemental Table 2.** Sensitivity, specificity, PPV, NPV for identifying renal cell carcinoma (excludes 11 non‐diagnostic/parenchyma biopsy).
