# Supplementary material for: Contemporary assessment of diagnostic performance and histologic concordance of renal mass biopsy with surgical pathology
Source: BJUI Compass. 2025 Nov 6;6(11):e70104. doi: 10.1002/bco2.70104 (PMC12591661; doi:10.1002/bco2.70104)
Supplement: Supplementary file 1 — Supplemental Table 1. Sensitivity, specificity, PPV, NPV for identifying renal cell carcinoma (includes 11 non‐diagnostic/parenchyma biopsy). Supplemental Table 2. Sensitivity, specificity, PPV, NPV for identifying renal cell carcinoma (excludes 11 non‐diagnostic/parenchyma biopsy). [file BCO2-6-e70104-s001.docx]

**Supplemental File**

Contents:

1. **Supplemental Table 1.** Sensitivity, specificity, PPV, NPV for identifying renal cell carcinoma (includes 11 non-diagnostic/parenchyma biopsy)
2. **Supplemental Table 2.** Sensitivity, specificity, PPV, NPV for identifying renal cell carcinoma (excludes 11 non-diagnostic/parenchyma biopsy)

**Supplemental Table 1.** Sensitivity, specificity, PPV, NPV for identifying renal cell carcinoma (includes 11 non-diagnostic/parenchyma biopsy)

|  |  | **Surgical Path** | |  |
| --- | --- | --- | --- | --- |
|  |  | Non-RCC | RCC | **Total** |
| **Biopsy Path** | Non-RCC | 10 | 14 | 24 |
|  | RCC | 4 | 215 | 219 |
|  | **Total** | 14 | 229 | 243 |

Definition of RCC: on biopsy - any subtype of RCC AND oncocytic neoplasm; on nephrectomy - any subtype of RCC

Definition of Non-RCC: on biopsy - oncocytoma, parenchyma/non-diagnostic (11), clear papillary tumor, xanthogranulomatous pyelonephritis; on nephrectomy – hybrid oncocytic/chromophobe tumor (HOCT), no tumor, clear papillary tumor, oncocytoma, cavernosal hemangioma, lymphoproliferative disorder

**Abbreviations:** RCC – renal cell carcinoma

**Supplemental Table 2.** Sensitivity, specificity, PPV, NPV for identifying renal cell carcinoma (excludes 11 non-diagnostic/parenchyma biopsy)

|  |  | **Surgical Path** | |  |
| --- | --- | --- | --- | --- |
|  |  | Non-RCC | RCC | Total |
| **Biopsy Path** | Non-RCC | 9 | 4 | 13 |
|  | RCC | 4 | 215 | 219 |
|  | Total | 13 | 219 | 232 |

Definition of RCC: on biopsy - any subtype of RCC AND oncocytic neoplasm; on nephrectomy - any subtype of RCC

Definition of Non-RCC: on biopsy - oncocytoma, clear papillary tumor, xanthogranulomatous pyelonephritis; on nephrectomy – hybrid oncocytic/chromophobe tumor (HOCT), no tumor, clear papillary tumor, oncocytoma, cavernosal hemangioma, lymphoproliferative disorder

**Abbreviations:** RCC – renal cell carcinoma
